# The Epidemiology, Clinical, and Economic Burdens of Respiratory Syncytial Virus Infections Amongst Hospitalized Children Under 5 Years of Age in Jordan: A National Multi-Center Cross-Sectional Study

**DOI:** 10.3390/v16121867

**Published:** 2024-11-30

**Authors:** Munir Abu-Helalah, Samah F. Al-Shatnawi, Mohammad Abu Lubad, Enas Al-Zayadneh, Hussein Jdaitawi, Mea’ad Harahsheh, Montaha AL-Iede, Omar Nafi, Ruba Yousef, Ihsan Almaaitah, Mai Ababneh, Toqa AlZubi, Rand Abu Mahfouz, Heba Adaylah, Hamzeh AlHajaj, Mohammad Al Tamimi, Simon B. Drysdale

**Affiliations:** 1 Department of Family and Community Medicine, Faculty of Medicine, Jordan University, Amman 11942, Jordan; 2 Public Health Institute, The University of Jordan, Amman 11942, Jordan; 3 Department of Clinical Pharmacy, Faculty of Pharmacy, Jordan University of Science and Technology, Irbid 22110, Jordan; sfshatnawi@just.edu.jo; 4 Department of Microbiology and Pathology, Faculty of Medicine, Mutah University, Karak 61710, Jordan; abu_lubbad@yahoo.com; 5 Department of Pediatrics, Faculty of Medicine, Jordan University, Amman 11942, Jordan; e.alzayadneh@ju.edu.jo (E.A.-Z.); m.al-iede@ju.edu.jo (M.A.-I.); 6 Department of Pediatrics, Ministry of Health, Princess Rahma Pediatrics Hospital, Irbid 21110, Jordan; rsv-team@yahoo.com; 7 School of Pharmacy, University of Jordan, Amman 11942, Jordan; mya9220222@ju.edu.jo; 8 Department of Pediatrics, Faculty of Medicine, Mutah University, Mutah 61710, Jordan; onafi2000@yahoo.com; 9 Medical Department, MENA Center for Research and Development, Amman 11942, Jordan; ruba.yousef1995@gmail.com; 10 Pediatrics Department, Zarqa Governmental Hospital, Zarqa 13110, Jordan; ihsanalmaaitah66@gmail.com (I.A.); mdhamzehhjj@gmail.com (H.A.); 11 MENA Center for Research and Development, Amman 11942, Jordan; mai.ababneh97@gmail.com (M.A.); toqaalzoubi66@gmail.com (T.A.); randabumahfouz@gmail.com (R.A.M.); hebaadaileh56@gmail.com (H.A.); 12 Independent Researcher, Zarqa 13133, Jordan; matmat1978@yahoo.com; 13 Oxford Vaccine Group, Department of Pediatrics, University of Oxford, Oxford OX1 2JD, UK; simon.drysdale@paediatrics.ox.ac.uk; 14 The NIHR Oxford Biomedical Research Centre, Oxford OX3 7JX, UK

**Keywords:** RSV, clinical, epidemiological, financial, Jordan

## Abstract

Respiratory syncytial virus (RSV) has been recognized as a highly important cause of morbidity and mortality among children and adults. A cross-sectional study at representative sites in Jordan was undertaken to provide an assessment of the epidemiology and health and economic burdens of RSV and influenza infections in Jordan amongst hospitalized children under 5 years old for the period between 15 November 2022 and 14 April 2023. This study involved 1000 patients with a mean age of 17.10 (SD: 16.57) months. Of these, half (*n* = 506, 50.6%) had positive results for RSV. Furthermore, 33% and 17.4% of the participants had positive results for RSV-B and RSV-A, respectively. The findings underscore the severity of RSV infections, where a significant proportion of the children experienced severe respiratory distress, which led to bronchiolitis and pneumonia. This study meticulously documented the clinical outcomes, including the need for intensive care, mechanical ventilation, and prolonged hospital stays. There was no statistically significant difference in the financial burdens between the RSV-positive and RSV-negative patients. This study revealed the urgent need for preventive measures to control the substantial burden of RSV among children under 5 years old in Jordan.

## 1. Introduction

Respiratory syncytial virus (RSV) was first identified in chimpanzees in 1955. Since then, it has been identified as the leading cause of viral bronchiolitis in human infants [1,2]. Moreover, it became a recognized highly important cause of morbidity and mortality among children and adults, particularly those with low immunity or chronic cardiac or respiratory illnesses [3].

It was estimated that 22% of all acute respiratory tract infections globally, the majority of which being in low-income countries, are due to RSV infections. In addition, recent global figures revealed that RSV leads to an estimated 34 million acute respiratory infections (ARIs), with an associated 3 million hospitalizations for severe RSV cases and almost 200,000 deaths among children aged 5 years or below [4,5].

There is scarce data on the burden of RSV in developing countries. A meta-analysis of the burden of RSV infection in Latin America was published in 2014 [6]. It revealed the large burden of RSV infections among infants, young children, and elderly adults. There was a high pooled percentage (41.5%) of RSV in lower respiratory tract infections in those 0–11 months old. In the elderly (≥60 years), there was also a fairly high pooled percentage of 12.6% [5].

Few hospital-based studies from Jordan have been published [7,8]. The first studies were conducted in 2010 and 2013 at one hospital in Amman, which included children younger than 2 years with acute respiratory symptoms. It showed that RSV was imposing a substantial burden on the healthcare sector. A total of 3168 hospitalized children with lower respiratory tract infections were included. More than 80% of them tested positive for one virus, with RSV being the most common virus detected (44%).

Another study from the same hospital included children up to the age of 5 years with the same criteria. Out of the 728 included subjects, 467 (64%) tested positive for RSV using RT-PCR [8].

These two studies are of great value; however, they had some limitations for current use in Jordan and there is a need for a more recent study for data beyond 2013. In addition to the need for more recent and robust data, there is a need for data from representative areas from the north, middle, and south of Jordan. More robust and representative data are needed for decision makers to invest in the prevention of RSV infections. Therefore, the details of hospitalized cases, complication rates, and direct and indirect costs at representative sites are of high need locally.

## 2. Materials and Methods

### 2.1. Study Design

The multi-center cross-sectional study design involved four study centers distributed in the central, northern, and southern regions of Jordan as follows: (1) Princess Rahma Hospital for Children, Irbid; (2) Zarqa Hospital, which serves the center and east of Jordan; (3) Jordan University Hospital, Amman; and (4) Al Karak Public Hospital, Al Karak.

Eligible patients who presented with respiratory symptoms in the inpatient clinics at the four study sites, according to below criteria. The recruitment of patients took place during weekdays and continued during weekends and holidays to support diversity in the access to enrollment.

### 2.2. Inclusion Criteria

Patients that permanently resided in the study areas and hospitalized with acute respiratory infection according to the below definition and criteria.

### 2.3. Case Definition [9]

Diagnosis of acute respiratory infection, which was defined as “an illness presenting with one or more of the following symptoms for less than 7 days: fever, cough, earache, nasal congestion, rhinorrhea, sore throat, vomiting after coughing, crackles, and labored, rapid or shallow breathing”.

### 2.4. Study Population

Children < 5 years of age admitted to the study sites with the following:

(1) At least one sign of an acute infection (temperature ≥ 38 °C or <35.5 °C, abnormal white blood cell [WBC] count or abnormal differential).
(2) Diagnosis of acute respiratory infection as defined above.

### 2.5. Exclusion Criteria

Not a permanent resident of Jordan.

### 2.6. Sample Collection and Processing

- Nasopharyngeal (NP) specimens were collected from each patient who met the inclusion criteria and consented to this study. An NP swab was taken and then a Multiplex viral reverse transcription polymerase chain reaction (RT-PCR) was performed on each nasopharyngeal specimen.
- Polymerase chain reaction (PCR) was used to diagnose cases with RSV at the included sites.
- RSV-positive samples were further analyzed via subtyping for RSV-A and RSV-B.

### 2.7. Microbiology

#### Sample Collection and Transport

Flocked swabs with plastic shafts were used to collect nasopharyngeal specimens from each patient who met the inclusion criteria. The swabs were then inserted into sterile viral transport media (VTM) and immediately placed on refrigerant gel packs or held at 4 °C prior to transport to the laboratory on the day of collection. Upon arrival at the laboratory, the specimens were immediately processed. The whole genome was extracted from the specimens and stored at −80 °C for further analysis to identify the target viruses.

Multiplex RT-PCR (TaqPath™ Combo Kit-Thermofisher, Waltham, MA, USA) was performed on each nasopharyngeal specimen by testing for respiratory syncytial virus (RSV) using the QuantStudio 5 instrument (Applied Biosystem, Foster, CA, USA) according to the manufacturer’s instructions. RSV subtypes A and B were then detected using a VIASURE Real-Time Detection Kit (CerTest Biotec S.L., Zaragoza, Spain). The RSV subtype RT-PCR cycles were as follows: 1 cycle for reverse transcription for 15 min at 45 °C, initial denaturation for 2 min at 95 °C, 45 cycles of denaturation for 10 s at 95 °C, and 45 cycles of annealing/extension for 50 s at 60 °C [10,11]. Samples were also analyzed for Influenza using the same kit. 

### 2.8. Power/Sample Size

We expected to enroll 1000 subjects who matched the above clinical criteria. According to previous studies, 2–25% of these subjects could have been positive for RSV [4,5]. Therefore, we expected 20% of the subjects to be positive for RSV when calculating the sample size. The recruitment took place during weekends (Saturdays) and holidays, as well as weekdays. Previous data from Jordan showed that up to 40% of hospitalized children younger than 5 years are positive for RSV. However, we used a more conservative estimate based on more recent epidemiological studies. Due to the higher incidence of RSV infection for the <2 years old age group, the sample was stratified into 0–2 and >2–5 years old. The pragmatic sample size of 1000 was based on the flow of patients with respiratory symptoms in the in- and outpatient clinics.

### 2.9. Statistical Methods

Statistical Package for the Social Sciences (SPSS) version 23 was used to analyze the data. Descriptive statistics (Student’s *t*-test and chi-squared test) were used to analyze and compare the categorical variables: demographic characteristics, including the patient characteristics; risk factors; pre-hospital antibiotic use; and vaccination details. Logistic regression analysis was used to identify the predictors of RSV positivity and predictors of complications.

### 2.10. Case Report Form (CRF)

Each study participant had a unique case ID throughout this study. The first part of the form included the inclusion criteria for this study, as described above. The parents/guardians of the eligible patients were asked whether they consented to take part in this study after explaining the study details. All were reassured that participation was voluntary and would not affect the service provided. There was a daily site report that counted the numbers of admissions; eligible patients; and included and excluded patients, including the reasons for not participating.

### 2.11. The Interview Forms Included Five Sections

1. Background, demographic, and societal data for patients and parents: sex, age, parents’ ages and educational statuses, special diet/milk, number of people in the household with age groups, patients or siblings attending a kindergarten, and parents or siblings with a history of asthma or eczema. Furthermore, a detailed smoking history was obtained, including the mother’s history of smoking during pregnancy; the father’s, mother’s, or other household members’ smoking (inside or outside the home); and the number of cigarettes per day or waterpipe per week.
2. Medical history, including birth history, existing medical conditions, and current regular medications: weight, height, birth weight, gestational age at birth, method of delivery, meconium-stained liquor, neonatal intensive care unit (NICU) admission and ventilation status, surfactant, breast feeding, asthma, cystic fibrosis, bronchopulmonary dysplasia, neuromuscular disease, congenital heart disease, other congenital disease, immunodeficiency, eczema (atopy), and other comorbidities. Any previous hospital admission with the diagnosis, duration, and dates were included. Vaccine statuses for Pneumococcal Conjugate Vaccine (PCV), flu, and SARS-CoV-2 for the patient, mother, father, and siblings were obtained. Regular medications and their indications. Medical history of RSV treatments during the current admission or previous admissions.
3. Presenting symptoms and signs: Symptoms and their duration prior to admission were included. Other clinical manifestations, such as cardiovascular manifestations, dehydration, wheezes, cyanosis, low activity level, hypoxia (SaO2 < 92%), tachypnea, pneumothorax/atelectasis, apnea > 10 s, subcostal/intercostal retractions, and nasal flaring.
4. Societal cost details were covered: number of relatives or caregivers who accompanied the patient, relationship of caregiver to the patient, transportation duration, methods and cost per trip, and number of trips for the other household members and the cost.

Income lost for the caregivers, and income lost by other family members due to the patient admission: whether the illness affected the family financially and details about the money source for covering the expenses or work loss due to the illness, such as cutting down on other expenses, using savings, borrowing, selling assets, donations from friends and relatives, and other.

5. Admission medical and financial details: complications, intensive care unit (ICU) admission, and outcomes at discharge. Medical and financial details: investigations, consumables, medications, hospital administrative cost, healthcare providers care and consultations, medical and financial data due to readmissions or revisits to the emergency or outpatients due to the same illnesses. Finally, medical care and financial data prior to admission, such as outpatient or emergency department details and their financial costs.
6. Laboratory findings, including WBC and differential, blood gas, PCR results, chest X-ray findings on arrival, pharyngeal swab, bacterial coinfection, fungal coinfection, and other.
7. Healthcare prior to hospitalization at public or private hospitals and clinics and financial cost of the provided care. This also covered care provided at the emergency department at the same hospital prior to admission.

All utilized medicines during the admission, including the name, route, dose, frequency, number of days during admission, and final cost per medication were recorded for every patient. This included all discharge medications, if prescribed.

## 3. Results

### 3.1. Inpatient Data

There were 3580 admissions at study sites during recruitment phase between 15 November 2022 and 14 April 2023. 1755 children were screened. 1008 were eligible for the study; 8 of them were not included due to failure of parents to sign the consent form. A total of 1000 individuals who met the criteria were enrolled in this study.

**Figure 1:**
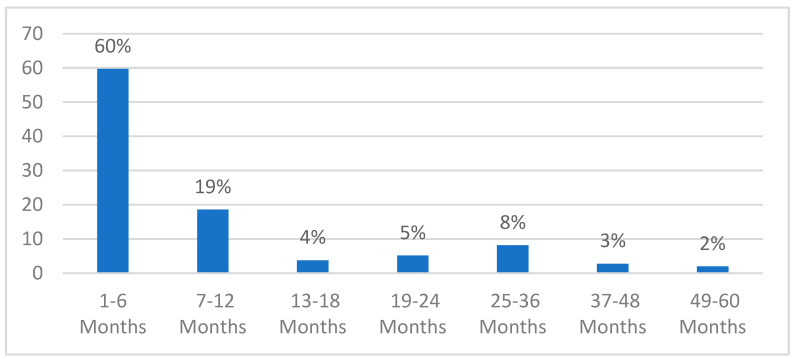
RSV positivity by age group.

The mean age was 17.10 (SD: 16.57) months and the median age was 9.68 (Q1–Q3: [3.13–29.83]) months. Approximately 59% of our sample patients were male and 68% of them were younger than 2 years. Half (*n* = 506, 50.6%) of the sample had positive results for RSV; 33% (*n* = 332) and 17.4% (*n* = 174) of the participants had positive results for RSV-B and RSV-A, respectively. The highest positivity rates were reported for children within the first 6 months of age, followed by 7 to 12 months. The positivity rates were less than 10% for children aged 2 to 5 years (Figure 1). Moreover, 9.9% of the participants were positive for influenza. Data will be published in a separate report. Positivity for influenza was included in the regression analysis for RSV positivity and for presence of complications. Positivity rate by month for the recruitment period between November 2022 and April 2023 are shown in Supplementary Table S1. The highest positivity rate was reported in November, while the lowest positivity rates were reported in March and April. 

In the examination results of the socioeconomic and medical histories of the RSV-positive and -negative individuals using chi-squared analysis shown in Supplementary Table S2, several significant disparities came to light. The percentage of RSV-positive patients was statistically higher in the north region compared with the middle and south (66.80% versus 46.20% and 43.20%, respectively; *p*-value < 0.001). 

The data provided in Table 1 illustrates the distribution of various symptoms across the subjects of this study. When examining the clinical symptoms, it was observed that the RSV-positive cases had statistically higher rates of cough and hypoxia or cyanosis compared with those who tested negative for RSV (99.4% versus 92.9%, *p*-value < 0.001, and 32.6% versus 23.3%, *p*-value = 0.001, respectively). The complications during hospitalization and other clinical outcomes are shown in Table 1. A high rate of at least one complication (74.3%) was documented in the RSV-positive cases, while 64.8% of RSV-negative cases had at least one complication during admission (*p* < 0.001).

Dehydration as a complication was more prevalent among the RSV-positive patients than the RSV-negative patients, with rates of 25.9% versus 20.6%, respectively (*p*-value = 0.050). Other clinical indicators, such as the presence of hypoxia below 92%, were also significantly higher among the RSV-positive patients than the RSV-negative patients (Table 2).

### 3.2. Predictors of RSV Positivity

Table 3 shows the investigated factors that were associated with RSV positivity across all age groups. The participants that resided outside the capital, such as in Irbid and Zarqa, had a much higher probability of testing positive for RSV than those in Amman, which is the capital of Jordan. Furthermore, age showed a slight but significant correlation, i.e., a minor decrease in the chance of being RSV-positive as age increased. The individuals that tested positive for influenza had lower odds of being RSV positive compared with those that tested negative. Notably, symptoms such as a cough, sore throat, and nasal congestion, as well as certain comorbidities, such as congenital heart disease and eczema, were associated with increased RSV-positivity odds. In patients under the age of two years, similar trends were noted. Overcrowding was also found to be a significant predictor of RSV positivity.

### 3.3. Predictors of Complications

Further regression analysis was conducted for the predictors of the presence of at least one complication, with the results shown in Table 4. This included: Definition of one complication: mortality, bacterial coinfection, respiratory distress requiring Oxygen need either invasive or non-invasive, pneumonia, cardiovascular complications including heart failure or bradycardia or other cardiovascular complications, respiratory failure. 

First, compared with the RSV-negative patients, RSV-positive patients showed a significantly greater probability for complications (*p* < 0.001). Furthermore, the individuals with asthma had significantly higher risks for complications than those without asthma (OR = 3.8, *p* = 0.02).

For the participants younger than two years, the patients with asthma had a considerably higher risk of complications, which emphasizes the need for controlling comorbid diseases. Surprisingly, attending kindergarten or day care reduced complications, which was probably due to a greater immunity from early pathogen exposure. RSV infection remained a significant predictor of complications, where positive cases showed markedly higher odds of adverse outcomes (*p* < 0.001).

RSV-positive patients had higher risks of pneumonia and ICU admission compared with RSV-negative patients (*p* < 0.05).

Furthermore, preterm birth increased the chance of ICU admission, which underscores premature infants’ susceptibility and the need for specialized neonatal care interventions. Moreover, the presence of comorbidities and siblings’ attendance at kindergarten or day care facilities were identified as risk factors for ICU admission, which highlights the necessity of preventive efforts and tailored treatments for vulnerable groups. On the other hand, a positive flu vaccination status for patients revealed a protective impact, where it lowered the probability of ICU admission and demonstrated the value of preventive healthcare interventions in reducing the disease severity.

### 3.4. Length of Hospital Stay

The mean duration of a hospital stay was 4 days for RSV-positive and -negative patients. Regression analyses were conducted to identify the predictors of long hospital stays across different age groups. From the analysis that included all age groups, the following factors were found to be significant in terms of lengthening the hospital stays: bacterial coinfection (β = −0.104, *p* < 0.001), preterm birth status (β = −0.059, *p* = 0.025), height (β = −0.114, *p* = 0.032), the presence of meconium-stained liquor (β = 0.057, *p* = 0.027), asthma (β = 0.057, *p* = 0.030), and the presence of other comorbidities (β = −0.109, *p* < 0.001).

### 3.5. Financial Data

The financial analysis shown in Table 5 provides a detailed breakdown of the costs associated with the hospital stays and medical care. The results reveal a variation in the costs across different components and units, and it highlights the financial impact of various aspects of healthcare, including the staff, medication, diagnostic, and other miscellaneous expenses.

The total medical cost, including medications, consumables, diagnostics, and other components, had a mean of JOD 545.45. The overall cost, which included direct medical, nonmedical, and indirect costs, had a mean of JOD 615.95.

The comparison with RSV-positive cases offered insights into specific cost implications for this subgroup of patients. The estimated mean of the overall cost for the RSV-positive cases was JOD 584.68, which was lower than for the RSV-negative patients, with a medical cost of JOD 647.98, but this was not statistically significant. However, when the median was calculated, both groups had similar total costs (JOD 373).

The medical expenses for the RSV-positive cases had a mean of JOD 516.9, and the mean hospital stay time was 4.01 days. The average cost of an ICU stay for the RSV-positive cases was JOD 39. It is noteworthy that the cost of readmission was significant only for RSV-positive cases, with a mean cost of JOD 50.45, while the RSV-negative cases showed zero cost for readmission. The overall cost, which included direct medical, nonmedical, and indirect costs, was slightly higher for the RSV-negative cases, but the difference was not statistically significant. It is crucial to consider these findings in the context of healthcare resource allocation and patient outcomes. The chi-squared test results indicate significant associations between the RSV results and the financial impact on other family members, as well as the overall financial impact on the family.

The two-sample *t*-test results show that there was no significant difference in the transportation costs, time lost by caregivers, or income lost due to illness between the RSV-negative and RSV-positive cases. The means and standard deviations for these financial factors were comparable in both groups.

### 3.6. Comparing RSV-A and RSV-B

In our study, RSV-A was shown to be less frequently identified and was associated with some features that were suggestive of less severe disease: for those under two years old, only 18.9% (*n* = 14) and 39.2% (*n* = 29) of those with RSV-A and RSV-B required ICU admission, respectively; for those between two and five years old, 23% (*n* = 5) and 9.1% (*n* = 2) of those with RSV-B and RSV-A required ICU admission, respectively. Interestingly, there was a statistically significant longer length of hospitalization for those children with RSV-A (3.73 days) compared with RSV-B (3.27 days) (*p* = 0.03). However, the overall financial costs did not differ significantly between the RSV-A and RSV-B cases.

## 4. Discussion

Our results reveal a high prevalence of RSV positivity (*n* = 506, 50.6%) amongst children less than 5 years old that presented with respiratory symptoms to inpatient settings. Furthermore, 33% (*n* = 332) and 17.4% (*n* = 174) of the participants had positive results for RSV-B and RSV-A, respectively.

Our figures show a higher prevalence of RSV positivity when compared with previous studies from Jordan and other parts of the world. The local increase in RSV burden in Jordan post-COVID-19 is consistent with the global trend in RSV burden. Upon the release of the COVID-19 control measures, data from different regions show a delayed RSV outbreak, with greater numbers of infected patients found in several countries, such as Israel, Australia, South Africa, New Zealand, France, United States, and Japan [12]. A recent study from Sri Lanka showed a much lower prevalence of RSV positivity of 28% in children younger than five years [13]. The proportions of RSV-A and RSV-B were consistent with our finding, where 72.14% of RSV patients had RSV-B. Another study [14] from Norway revealed that 40% of the enrolled 1096 children were infected with RSV. The differences seen between our study and the latter two studies may reflect the different testing techniques (the Sri Lankan study tested for RSV using the less sensitive method of immunofluorescence, whereas all samples in our study were tested using RT-PCR), differing inclusion criteria and definitions of respiratory illness, and different proportions in the varying age bands of participants (e.g., having more infants under one year old was likely to increase the numbers in hospital, and therefore, the RSV-positivity rate). In addition, the timeframes the studies were carried out over may have impacted the results. The limited exposure to RSV during winter 2020–2021 probably resulted in a cohort of young children in Jordan without a natural immunity to RSV, thereby raising the potential for increased RSV incidence, out-of-season activity, and health service pressures when measures to restrict SARS-CoV-2 transmission were relaxed. In addition, the limited availability of monoclonal antibodies (Palivizumab), mainly in the public sector in Jordan, for high-risk children may have increased the rate of hospitalization and complications from RSV infections.

Although there remains debate about whether RSV-A or RSV-B results in more severe disease, RSV-A was shown to result in more febrile illness in infants [15], which was one of the important features of the case definition in this study.

The risk factors for RSV disease in our population were similar to previous studies [16] and included younger age, multiple births, male sex, low socioeconomic status and parental education, attending day care, young siblings, parental smoking, and a family history of atopy or asthma.

Although the majority of children with RSV infection present with mild upper respiratory tract infections, our study included a large proportion of those with lower respiratory tract infections (48% had wheeze and 35% reported difficulty breathing). Many of the participants were clinically diagnosed with bronchiolitis or pneumonia. In addition, 6.2% of the infants with an RSV infection presented with apnea, which can be a life-threatening complication that necessitates close monitoring in a healthcare setting. When examining clinical symptoms, it was observed that the RSV-positive cases had statistically higher rates of cough, dehydration, and hypoxia or cyanosis compared with the RSV-negative cases. This was most likely because the participants who were recruited included more infants who were hospitalized. A larger proportion of these participants tested positive for RSV compared with those with other respiratory viruses.

For those under two years old, 18.9% and 39.2% of those who tested positive for RSV-A and RSV-B required ICU admission, respectively, whereas for those between two and five years old, only 23% and 9.1% of those who tested positive for RSV-B and RSV-A required ICU admission, respectively. These data are consistent with the assessment of the RSV burden in Canada [17], which revealed a significant burden of RSV hospitalizations in Canadian pediatric centers, particularly among children under 6 months old. This study found an increase in admissions during 2021–2022, with regional variation in the RSV activity timing. The proportions of ICU admissions varied significantly across age groups (range: 214 of 1249 admissions among patients aged 6–11 months [17.1%] to 58 of 188 admissions among patients aged 10–16 years [30.9%], *p* < 0.001).

Bacterial superinfections, such as bacterial pneumonia or ear infections, are relatively uncommon complications for RSV infected patients, when compared with other viruses such as influenza A or measles. [18,19] In our study, 1.2% (*n* = 6) of individuals with RSV positivity experienced bacterial or fungal coinfection. The same was found in a previous 9-year prospective study [20] in the USA of 1706 children hospitalized with acute respiratory illnesses, where 565 children had documented RSV infections. A subsequent bacterial infection rarely developed in those with an RSV lower respiratory tract disease. This is also consistent with recent data from Illinois, USA [21].

Children with a history of asthma may experience exacerbations of their respiratory symptoms during and following an RSV infection. The virus can trigger inflammation and bronchoconstriction, which leads to increased wheezing and shortness of breath in children with asthma. Managing an RSV infection in asthmatic children requires a comprehensive approach that includes bronchodilators and anti-inflammatory medications. In our study, a previous diagnosis of asthma was associated with an increased risk of complications with an RSV infection. This is consistent with other studies that found that a personal or family history of atopy was associated with more severe RSV [16,22].

Overall, the epidemiology, clinical presentation, and complication data for children infected with RSV in this study are similar to previously reported data from across the MENA region and wider world. This is reassuring and suggests that the newly available RSV monoclonal antibody (nirsevimab) and maternal vaccine (ABRYVESO) would likely have similar beneficial effects to those already demonstrated in real-world settings [23].

It is important to recognize the limits of this study and proceed with caution when interpreting these data and establishing causal correlations. Research on nosocomial RSV infection, such as that carried out by Altamimi et al. (2019), emphasized the importance of taking into account outside variables that may affect the study outcomes and offers a framework for comprehending the difficulties involved in infection control [24,25]. In their study, Rothman et al. (2012) emphasized the necessity of age-specific factors when interpreting regression results by specifically pointing out the importance of taking respiratory syncytial virus hospitalizations among children under the age of two years into account [26,27,28].

### Overview of Financial Analysis Results

Through rigorous financial research, the economic impact of the respiratory syncytial virus (RSV) in Jordan was comprehensively explored. This study carefully dissected the expenses related to hospital stays and medical care by considering expenses such as staff, medical care, and hospitalization costs.

The lack of statistical significance in the financial burden between RSV-positive and RSV-negative patients could have been due to the presence of other infections, such as influenza. This will be analyzed further in future reports. However, our findings are consistent with results from other developing countries [29,30]. A study from Malawi revealed that for infants who received care at a referral hospital, the cost per episode in which RSV was detected was comparable with that of other episodes of respiratory illnesses where RSV was not detected [31]. The study by Al-Eyadhy et al. shed important light on the financial cost of respiratory viruses in people who have asthma, COPD, or asthma–COPD overlap syndrome. Differences in the financial burden could have been due to access to management. The financial burden should be based on local data in health economics decisions related to the local management or prevention of RSV infections. The findings may have relevance for Jordanian healthcare planning and policies since they advance our knowledge of the financial effects of viral respiratory illnesses in susceptible populations [32].

Critical factors that determined the total cost of respiratory syncytial virus (RSV) infections were revealed by a regression analysis, the results of which are consistent with the body of research on disease severity and healthcare consumption. Studies such as Rothman et al. (2012) highlighted the significance of comprehending hospitalizations among young children with respiratory illnesses, including RSV, to go further into the influence of disease severity on expenditures [26,27,28]. Furthermore, as illustrated by Al-Eyadhy et al. (2017), analyzing the financial impact of respiratory viruses on individuals with long-term respiratory disorders might provide information on the wider financial ramifications of RSV [32].

The results for this age group highlight how important factors such as illness severity, comorbidities, and bacterial coinfection are. The clinical features and effects of respiratory infections were clarified by a study by Alshehri et al. (2020) on pneumonia in children in Saudi Arabia, which offers context for comprehending the predictors found in our analysis [29,33].

Similar to our findings, Al-Mutairi et al. (2016) investigated the economic impact of RSV infections by taking into account variables such place of the residence, preterm delivery, and particular diagnoses, which laid the groundwork for comprehending the complex predictors reported in this analysis [30,34].

This study has a representative sample from different regions in Jordan, however it has several limitations. The first one is that data was collected between November and April, which is the RSV season in Jordan. Data on the annual incidence and seasonal variations could not be obtained. Also, the kit used was assessing influenza and RSV. The presence of other infections among the RSV positive and negative cases might have affected the differences between RSV positive and RSV negative cases. This was handled partially by including positivity against influenza in the regression analysis and was a not a predictor for RSV positivity or presence of complications. 

## 5. Conclusions

This national multi-center cross-sectional study in Jordan offers a robust exploration of the epidemiology, health impacts, and economic burdens of RSV among children under 5 years of age. This comprehensive study aimed to provide a detailed understanding of the prevalence, risk factors, clinical outcomes, and associated economic implications of RSV infections in this vulnerable population.

This study revealed a substantial burden of RSV among children under 5 years in Jordan, with a high prevalence observed across multiple healthcare centers.

Financial data from this study can provide the basis for future health economics models for the prevention of RSV infection for different age groups and for children at high risk of complications. This would facilitate cost-effective decisions based on local data.

Overall, the insights gained from this research have far-reaching implications by providing a basis for evidence-based healthcare policies, preventive measures, and resource allocation strategies to address the significant challenges posed by RSV infections in the pediatric population. This study not only adds valuable knowledge to the global understanding of RSV but also serves as a model for future research and public health initiatives in combating respiratory infections in young children.

## Figures and Tables

**Table 1:** Presence of symptoms by RSV positivity.

		RSV Result				
		Negative		Positive		
Symptoms		Count	Column N%	Count	Column N%	*p*-Value *
Fever	No	8	1.6%	4	0.8%	0.23
	Yes	486	98.4%	502	99.2%	
Cough	No	35	7.1%	3	0.6%	<0.001
	Yes	459	92.9%	503	99.4%	
Sore throat	No	374	75.7%	442	87.4%	<0.001
	Yes	120	24.3%	64	12.6%	
Rhinorrhea	No	172	34.8%	261	51.6%	<0.001
	Yes	322	65.2%	245	48.4%	
Nasal congestion	No	237	48.0%	309	61.1%	<0.001
	Yes	257	52.0%	197	38.9%	
Poor feeding	No	219	44.3%	288	56.9%	<0.001
	Yes	275	55.7%	218	43.1%	
Hypoxia (SaO2 < 92%)/cyanosis	No	379	76.7%	341	67.4%	<0.001
	Yes	115	23.3%	165	32.6%	
Breathlessness	No	289	58.5%	283	55.9%	0.41
	Yes	205	41.5%	223	44.1%	
Respiratory crackles	No	225	45.5%	209	41.3%	0.18
	Yes	269	54.5%	297	58.7%	
Apnea > 10 s	No	465	94.1%	473	93.5%	0.67
	Yes	29	5.9%	33	6.5%	
Wheezing	No	271	54.9%	293	57.9%	0.33
	Yes	223	45.1%	213	42.1%	
Low activity level	No	208	42.1%	255	50.4%	0.01
	Yes	286	57.9%	251	49.6%	
Tachypnea	No	304	61.5%	354	70.0%	0.005
	Yes	190	38.5%	152	30.0%	
Post-tussive vomiting	No	323	65.4%	336	66.4%	0.73
	Yes	171	34.6%	170	33.6%	
Other symptoms	No	379	76.7%	456	90.1%	<0.001
	Yes	115	23.3%	50	9.9%	

* Chi-squared test, statistically significant at *p* < 0.05.

**Table 2:** Descriptive statistics of clinical findings by RSV positivity.

		PCR RSV Result				
		Negative		Positive		
		Count	Column N%	Count	Column N%	*p*-Value *
Chest X-ray infiltrate	No	175	35.4%	178	35.2%	0.94
	Yes	319	64.6%	328	64.8%	
White blood cell count (×10^9^/L)	<4.0	9	1.8%	4	0.8%	0.25
	>10.0	307	62.1%	321	63.4%	
	4 to 10	176	35.6%	181	35.8%	
	NA	2	0.4%	0	0.0%	
Other clinical manifestations:	No	59	11.9%	40	7.9%	<0.001
	Yes	435	88.1%	466	92.1%	
Cardiovascular complications ^	No	490	99.2%	500	98.8%	0.55
	Yes	4	0.8%	6	1.2%	
Low activity level	No	336	68.0%	366	72.3%	0.14
	Yes	158	32.0%	140	27.7%	
Apnea > 10 s	No	490	99.2%	500	98.8%	0.55
	Yes	4	0.8%	6	1.2%	
Dehydration	No	392	79.4%	375	74.1%	0.05
	Yes	102	20.6%	131	25.9%	
Hypoxia (SaO2 < 92%)	No	404	81.8%	362	71.5%	<0.001
	Yes	90	18.2%	144	28.5%	
Subcostal/intercostal retractions	No	296	59.9%	273	54.0%	0.06
	Yes	198	40.1%	233	46.0%	
Wheezes	No	241	48.8%	193	38.1%	0.001
	Yes	253	51.2%	313	61.9%	
Tachypnea—other clinical	No	253	51.2%	233	46.0%	0.10
	Yes	241	48.8%	273	54.0%	
Cyanosis—other clinical	No	469	94.9%	453	89.5%	0.001
	Yes	25	5.1%	53	10.5%	
Pneumothorax/atelectasis	No	492	99.6%	505	99.8%	0.55
	Yes	2	0.4%	1	0.2%	
Acute respiratory distress	No	349	70.6%	369	75.9%	0.42
	Yes	145	29.4%	137	27.1%	
Nasal flaring	No	487	98.6%	495	97.8%	0.37
	Yes	7	1.4%	11	2.2%	
Required ICU admission	No	448	90.7%	456	90.1%	0.76
	Yes	46	9.3%	50	9.9%	
Non-invasive oxygen ventilation needed	No	462	93.5%	479	94.7%	0.44
	Yes	32	6.5%	27	5.3%	
Invasive oxygen ventilation needed	No	492	99.6%	499	98.6%	0.10
	Yes	2	0.4%	7	1.4%	

* Based on Chi-Square analysis; ^ cardiovascular complications including heart failure, bradycardia, pulmonary hypertension etc.

**Table 3:** Binary logistic regression analysis of factors associated with a positive RSV result across all age groups.

	B	*p*-Value	Odds Ratio	95% C.I
City		<0.001		
Irbid	2.62	<0.001	13.69	5.71–32.79
Zarqa	2.43	<0.001	11.32	4.87–26.32
Karak	0.82	0.02	2.27	1.13–4.54
Amman	Reference			
Age (months)	−0.07	<0.001	0.94	0.92–0.95
Influenza				
Positive	−2.15	<0.001	0.12	0.06–0.24
Negative	Reference			
Cough symptom present				
Yes	2.24	0.001	9.37	2.43–36.17
No	Reference			
Sore throat symptom present				
Yes	−0.76	0.03	0.47	0.23–0.94
No	Reference			
Nasal congestion symptoms				
Yes	1.03	0.001	2.81	1.51–5.24
No	Reference			
Nasal congestion duration	−0.12	<0.001	0.88	0.83–0.94
Duration of respiratory crackles in days	0.08	0.02	1.08	1.01–1.15
Duration of post-tussive symptoms	0.11	0.03	1.12	1.01–1.24
Total number in the household	0.10	0.04	1.10	1–1.2
Overcrowding				
Yes	−0.68	0.04	0.51	0.27–0.98
No	Reference			
Congenital heart disease				
Yes	−1.02	0.02	0.36	0.16–0.83
No	Reference			
Eczema (atopy)				
Yes	−1.48	0.02	0.23	0.06–0.82
No	Reference			
Presented as acute respiratory distress				
No	−0.64	0.009	0.53	0.33–0.85
Yes	Reference			
Presence of respiratory acidosis				
Yes	1.01	0.006	2.76	1.34–5.69
No	Reference			

**Table 4:** Binary logistic regression analysis of the factors associated with the presence of complications across all age groups.

	B	*p*-Value	Odds Ratio	95% C.I
RSV				
Positive RSV	6.361	<0.001	578.699	198.705–1685.373
Negative RSV	Reference			
City		<0.001		
Irbid	−3.60	<0.001	0.03	0.008–0.095
Zarqa	−1.97	0.003	0.14	0.039–0.504
Karak	−2.96	<0.001	0.05	0.015–0.179
Amman	Reference			
Asthma				
Yes	1.34	0.02	3.80	1.263–11.422
No	Reference			
Patient attending kindergarten or day care				
Yes	−2.27	0.001	0.10	0.026–0.419
No	Reference			
Temperature ≥ 38 °C	−2.18	0.03	0.11	0.017–0.763
Temperature < 35 °C	−0.30	0.85	0.74	0.034–16.021
None	Reference			
White blood cell count (×10^9^/L)		0.005		
<4	−4.42	0.001	0.01	0.001–0.175
>10	−0.24	0.43	0.79	0.444–1.408
4 to 10	Reference			
Chest X-ray infiltrate				
Yes	3.26	< 0.001	26.07	14.241–47.735
No	Reference			

**Table 5:** Descriptive financial statistics by RSV results.

Items	RSV Positive	N	Mean	Std. Deviation	*p*-Value *
Cost of hospital stay	No	494	134.41	198.36	0.01
	Yes	506	102.30	180.25	
Length of ward stay (days)	No	494	3.61	2.38	0.21
	Yes	506	3.42	2.27	
Caregiver cost	No	494	192.63	168.64	0.90
	Yes	506	191.31	169.68	
Total medications cost	No	494	47.02	183.37	0.03
	Yes	506	27.71	72.79	
Total consumables cost	No	494	51.72	103.12	0.222
	Yes	506	61.41	143.69	
Total diagnostics cost	No	494	114.12	170.76	<0.001
	Yes	506	62.98	91.54	
Total cost of ward	No	494	91.53	105.85	<0.001
	Yes	506	59.96	76.52	
Total cost of ICU	No	494	33.80	147.23	0.54
	Yes	506	39.56	149.77	
Total cost of isolation	No	494	9.08	64.64	0.049
	Yes	506	2.79	29.97	
	Yes	506	1.08	13.64	
Cost of readmission	No	494	0.00	0.00	<0.001
	Yes	506	50.45	201.40	
Cost of EM same visit	No	494	27.03	42.52	<0.001
	Yes	506	16.44	44.36	
Transportation	No	488	38.94	51.98	0.10
	Yes	504	33.58	48.99	
Family loss of income	No	493	34.92	110.74	0.92
	Yes	506	34.29	80.49	
Total medical cost **	No	494	574.66	648.07	0.17
	Yes	506	516.94	670.03	
Overall cost ***	No	494	647.98	697.87	0.16
	Yes	506	584.68	713.87	

* *t*-test, statistical significance <0.05. ** The total medical cost included the costs of medications, consumables, labs/diagnostics, the hospital stay, service prior to admission, and readmission. *** The overall cost included the direct medical, indirect medical, and societal costs (cost of transportation, and income lost due to hospital stay/visits).

## Data Availability

The original contributions presented in the study are included in the article/Supplementary Material, further inquiries can be directed to the corresponding author.

## Supplementary Materials

The following supporting information can be downloaded at https://www.mdpi.com/article/10.3390/v16121867/s1. Supplementary Table S1: RSV positivity by Month; Supplementary Table S2: Investigating demographic Factors Associated with RSV results.
