# Supplementary material for: The Epidemiology, Clinical, and Economic Burdens of Respiratory Syncytial Virus Infections Amongst Hospitalized Children Under 5 Years of Age in Jordan: A National Multi-Center Cross-Sectional Study
Source: Viruses. 2024 Nov 30;16(12):1867. doi: 10.3390/v16121867 (PMC11680125; doi:10.3390/v16121867)
Supplement: Supplementary file 1 [file viruses-16-01867-s001.zip › viruses-3255125-supplementary.pdf]

Supplementary Table S1 RSV positivity by Month

			RSV result		Total	
			Negative	Positive		p-value*
**Date- month**	Nov-2022	Count	95	267	362	**< 0.001**
		% within Month	26.24%	73.76%	100%	
	Dec-2022	Count	280	307	597	
		% within Month	47.70%	52.3%	100%	
	Jan-2023	Count	197	33	230	
		% within Month	85.65%	14.35%	100%	
	Feb-2023	Count	74	2	76	
		% within Month	97.37%	2.63%	100%	
	Mar-2023	Count	77	0	77	
		% within Month	100%	0.0%	100%	
	Apr-2023	Count	5	0	5	
		% within Month	100%	0.0%	100%	

***Chi-square, statistically significant at P < 0.05.**

Supplementary Table S2: Investigating demographic Factors Associated with RSV results.

**Characteristic**		RSV				**p-value***
		**Negative (N=494)**		**Positive (N=506)**		
		Count	Row N %	Count	Row N %	
**Age in months, mean**			24.44		9.94	
**Age in months, median, [Q1-Q3]**		24.02, [7.79-38.16]		5.27, [2.2-11.05]		
**Gender, n (%)**						
**Female**		190	45.89%	224	54.11%	
**Male**		304	51.88%	282	48.12%	
**Region, n (%)**						**<0.001**
· **Middle**		269	53.80%	231	46.20%	
· **North**		83	33.20%	167	66.80%	
· **South**		142	56.80%	108	43.20%	
**City, n (%)**						**<0.001**
· **Amman**		171	68.40%	79	31.60%	
· **Irbid**		83	33.20%	167	66.80%	
· **Karak**		142	56.80%	108	43.20%	
· **Zarqa**		98	39.20%	152	60.80%	
**Living area, n (%)**						0.38
· **Rural**		155	47.40%	172	52.60%	
· **Urban**		339	50.37%	334	49.63%	
**Admission through, n (%)**						**0.003**
· **Emergency department referral**		54	47.37%	60	52.63%	
· **Emergency department without referral**		369	47.49%	408	52.51%	
· **General practitioner**		2	33.33%	4	66.67%	
· **Outpatient clinic**		67	68.37%	31	31.63%	
· **Pediatrician**		2	40.00%	3	60.00%	
**Preterm or Full term**	full term	418	49.58%	425	50.42%	0.79
	preterm	76	48.41%	81	51.59%	
**WGA (Weeks for Gestational Age)**	<29	2	100.00%	0	0.00%	0.69
	29-30	1	25.00%	3	75.00%	
	31-32	8	50.00%	8	50.00%	
	33-34	12	48.00%	13	52.00%	
	35-36	53	48.18%	57	51.82%	

	>=37	418	49.58%	425	50.42%	
**Delivery**	Caesarean section	240	51.50%	226	48.50%	0.13
	Vaginal delivery	254	47.57%	280	52.43%	
**Meconium-stained liquor**	NO	484	49.29%	498	50.71%	0.60
	YES	10	55.56%	8	44.44%	
**Neonatal Intensive Care Unit (NICU)**	NO	367	48.48%	390	51.52%	0.31
	YES	127	52.26%	116	47.74%	
**NICU ventilation**	NO	409	47.39%	454	52.61%	**0.001**
	YES	85	62.04%	52	37.96%	
**Surfactant Given**	NO	395	47.25%	441	52.75%	**0.002**
	YES	99	60.37%	65	39.63%	
**Breastfed**	Exclusive	174	50.43%	171	49.57%	**0.005**
	Mixed	158	49.22%	163	50.78%	
	NO	162	48.50%	172	51.50%	
**Mother smoking during pregnancy**	NO	471	49.47%	481	50.53%	0.83
	YES	23	47.92%	25	52.08%	
**Patient on special milk/diet**	NO	477	49.38%	489	50.62%	0.94
	YES	17	50.00%	17	50.00%	
**Overcrowding**	NO	404	48.27%	433	51.73%	0.11
	YES	90	55.21%	73	44.79%	
**Parents smoking cigarettes or Shisha**	NO	146	52.71%	131	47.29%	0.20
	YES	348	48.13%	375	51.87%	
**Smoking inside home by parents or other households**	NO	371	48.06%	401	51.94%	0.12
	YES	123	53.95%	105	46.05%	
**Patient regular medications**	NO	385	47.36%	428	52.64%	**0.007**
	YES	109	58.29%	78	41.71%	
**Highest completed education of mother**	Diploma school	46	51.69%	43	48.31%	**0.001**
	Primary school	36	33.03%	73	66.97%	
	Secondary school	239	48.68%	252	51.32%	
	University BSc	154	54.23%	130	45.77%	
	University Postgraduate	19	70.37%	8	29.63%	
**Highest completed education of Father**	Diploma school	40	50.00%	40	50.00%	**0.02**
	Primary school	53	38.97%	83	61.03%	
	Secondary school	240	48.48%	255	51.52%	
	University BSc	128	54.24%	108	45.76%	
	University Postgraduate	33	62.26%	20	37.74%	
**Chronic conditions:**						
**Asthma**	NO	469	49.47%	479	50.53%	0.85
	YES	25	48.08%	27	51.92%	
**Bronchopulmonary dysplasia**	NO	493	49.35%	506	50.65%	0.31
	YES	1	100.00%	0	0.00%	
**Congenital heart disease**	NO	464	48.43%	494	51.57%	**0.004**
	YES	30	71.43%	12	28.57%	
**Neuromuscular disease**	NO	483	48.99%	503	51.01%	**0.03**
	YES	11	78.57%	3	21.43%	
**Other comorbidities****	NO	415	46.73%	473	53.27%	**<0.001**
	YES	79	70.54%	33	29.46%	
**Cystic fibrosis**	NO	493	49.35%	506	50.65%	0.31
	YES	1	100.00%	0	0.00%	

**Other congenital disease*** **	NO	489	49.49%	499	50.51%	0.59
	YES	5	41.67%	7	58.33%	
**Immunodeficiency**	NO	494	49.40%	506	50.60%	
**Eczema (Atopy)**	NO	475	48.62%	502	51.38%	**0.001**
	YES	19	82.61%	4	17.39%	
**Patient Chronic Conditions**	NO	352	45.01%	430	54.99%	**<0.001**
	YES	142	65.14%	76	34.86%	
**Patient attending kindergarten or day care**	NO	428	47.08%	481	52.92%	**<0.001**
	YES	66	72.53%	25	27.47%	
**Parent with history of atopic eczema**	NO	442	48.10%	477	51.90%	**0.005**
	YES	52	64.20%	29	35.80%	
**Siblings attending kindergarten or daycare**	NO	391	48.69%	412	51.31%	0.37
	YES	103	52.28%	94	47.72%	
**Parent with history of asthma**	NO	455	49.67%	461	50.33%	0.60
	YES	39	46.43%	45	53.57%	
**Siblings with history of asthma**	NO	446	48.58%	472	51.42%	
	YES	48	58.54%	34	41.46%	
**Siblings <5 years living in the same household**	NO	230	56.37%	178	43.63%	**<0.001**
	YES	264	44.59%	328	55.41%	

*Chi-square test, statistically significant at P < 0.05.
** Other comorbidities: including congenital heart disease, Ventricular Septal Defect (VSD), Antenatally Diagnosed Congenital Pulmonary Airway Malformation (CPAM) Type 1 with extra lobar sequestration, and Complete Atrioventricular (AV) Canal, along with Atrial Septal Defect (ASD) and VSD leading to heart failure. Additionally, Down syndrome, Global Developmental Delay (GDD), and epilepsy. Other conditions include Reactive Airway Disease (RAD), Retinopathy of Prematurity (ROP), renal anomalies, seizures and Glucose-6-Phosphate Dehydrogenase Deficiency (G6PD).
***In terms of other congenital diseases, there is chest deformity, Gastroesophageal Reflux Disease (GERD), Pierre Robin Syndrome, horseshoe kidney, laryngomalacia, single kidney, single lung, hydrocephalus, and Vesicoureteral Reflux (VUR).
